# Toward a Holistic Approach in Aortic Stenosis Using Machine-Learning Algorithms

**DOI:** 10.1016/j.jacadv.2024.101134

**Published:** 2024-08-14

**Authors:** Anna Sannino, Lina Manzi

**Affiliations:** a Department of Advanced Biomedical Sciences, University of Naples Federico II, Naples, Italy; b Friede Springer-Centre of Cardiovascular Prevention, Charité Campus Benjamin Franklin, Berlin, Germany; c Cardiac Imaging Core Laboratory, Baylor Scott & White Research Institute, Plano, Texas

**Keywords:** aortic stenosis, artificial intelligence, echocardiography

**Figure:**
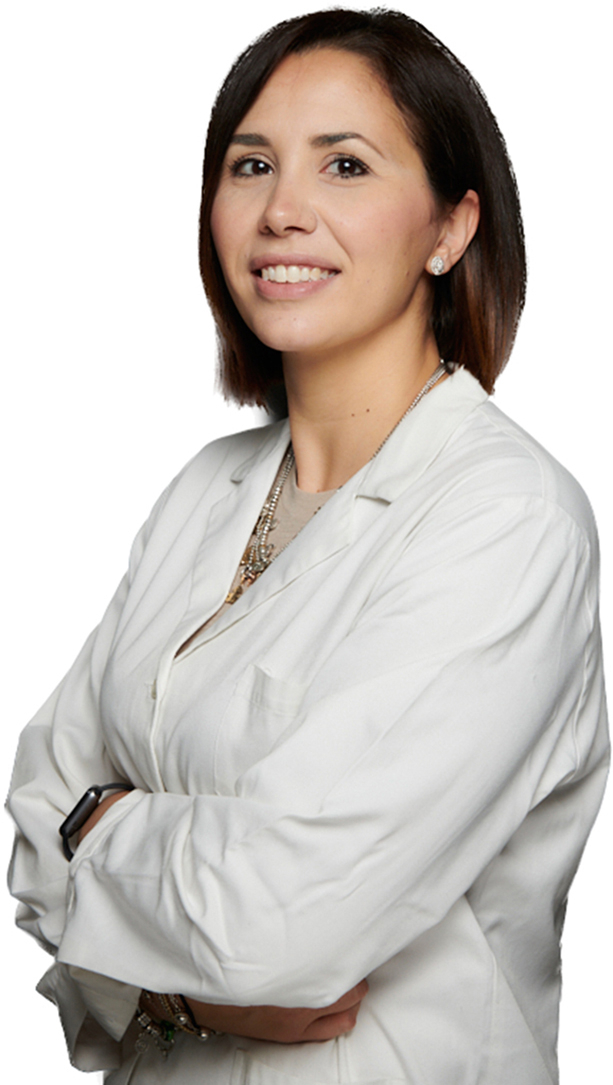

Aortic stenosis (AS) is the most common valvular disease requiring intervention in Europe and North America, and its prevalence is increasing due to the aging of the population.^1^^,^^2^ Current European and American guidelines recommend aortic valve replacement (AVR) in symptomatic patients with severe AS.^1^^,^^2^ Nonetheless, multiple evidences have shown that moderate AS or severe but asymptomatic AS are associated with unfavorable outcomes.^3^ Whether a redefinition in the timing and in the patient selection for intervention is needed, beyond grading and symptomatic status, remains to be answered.^4^ Patients with AS represent, in fact, a very heterogeneous population, in which an intricate relation between clinical, anatomical, and extra-valvular features plays a crucial role in driving the prognosis.

In this issue of *JACC: Advances*, Shimoni et al^5^ successfully developed a risk prediction model for patients with all grades of AS using machine-learning (ML) techniques to predict outcomes. The authors used clinical, echocardiographic, laboratory, and medication data to test a time-to-event model, the random survival forest, for AS prognosis. A SHapley Additive exPlanations method provided personalized risk assessment toward a composite outcome of AVR and mortality. The authors retrospectively evaluated 10,407 patients of the Kaplan Medical Center AS Registry who underwent echocardiography between 2008 and 2020 for model training and internal validation. Two additional patient cohorts were used for external validation. The proposed algorithm demonstrated good correlation between cohorts (area under the curve of 0.73 [95% CI: 0.72-0.74] and 0.74 [95% CI: 0.72-0.76]) as well as in the prediction of the composite endpoint at 1 and 5 years (area under the curve of 0.83 [95% CI: 0.80-0.86] and 0.83 [95% CI: 0.81-0.84]). From an initial model of 81 clinical and echocardiographic variables, 12 were selected and utilized to streamline and define the risk model. Interestingly, although the calculated risk score was directly proportional to AS severity, some patients with mild AS had a high risk score, whereas certain patients with severe AS showed a lower risk score. Although several ML studies have been published on diagnosis and grading of severe AS,^6^^,^^7^ none of these have ever validated a risk prediction model for patients across all grades of AS. By emphasizing the advantages of a risk predictor model across the spectrum of AS, Shimoni et al^5^ open the door to a personalized, patient-tailored approach that encompasses time and type of treatment as well as follow-up strategies before and after the intervention. It is worth noting, though, that their ML model only analyzed a snapshot of the disease course, not including longitudinal repeated measurement of the identified predictors. Additionally, other well-affirmed markers of AS progression and prognosis, such as brain natriuretic peptide or N-terminal fragment of brain natriuretic peptide, were not included in this analysis.^8^ Lastly, whether the proposed ML model could also be applied to patients with AS and reduced ejection fraction remains to be determined.^9^ Nevertheless, this study offers important additional points to be discussed.

First and foremost, the main concept that can be derived from this study is that AS patients must be evaluated in a very comprehensive manner. Grading AS based on echocardiography is not enough. If we look at the cardiac damage in AS, we will do better at predicting outcomes after AVR as compared to using AS severity measurements alone.^10^ In fact, a very recent study has shown that cardiac damage is very prevalent in all degrees of AS, starting with mild AS or stage 0. Importantly, it does not correlate with worsening AS severity but rather with the number of clinical comorbidities, further proving the crucial role of the clinical context.^11^

How and when the AS progresses to more severe stages are additional important questions that ML can help answer. Sánchez-Puente et al^12^ validated a ML model based on echocardiographic and clinical parameters to predict the progression of AS severity. Interestingly, according to their impact analysis, comparing the follow-up recommendations of their ML model with current European and American guidelines,^1^^,^^2^ the authors demonstrated that European guidelines resulted in an average of 2.2 “premature” visits per patient, American guidelines in an average of 1.3 “premature” visits per patient, whereas the ML system resulted only in an average of 1.1 “premature” visits per patient. Therefore, the ML system saved 13% and 49% of echocardiographic studies compared with the American and European guidelines. Considering that AS affected 2% to 5% of adults with an incidence rate of severe AS of 4% to 7% per year among persons >65 years of age,^13^^,^^14^ the proposed ML model could reduce unnecessary follow-ups, hospital workload, and the costs of the medical care system and, on the other hand, accelerate follow-up for those who will develop severe AS more rapidly.

Last but not least, this study from Shimoni et al further advances the topic of timing of intervention in AS. As mentioned, AS is associated with adverse outcomes across all grades.^15^^,^^16^ Steaming from these observations, several studies are now ongoing or were recently published to evaluate the optimal time of intervention in earlier stages of the disease. In the AVATAR trial (Aortic Valve Replacement Versus Conservative Treatment in Asymptomatic Severe Aortic Stenosis),^17^ the investigators evaluated the safety and efficacy of early AVR in severe AS and normal left ventricular function. Early AVR was associated with a reduction in the primary composite endpoint of all-cause death, acute myocardial infarction, stroke, or unplanned hospitalization for heart failure when compared to conservative treatment. Similar results have been reported in the RECOVERY trial (Early Surgery or Conservative Care for Asymptomatic Aortic Stenosis),^18^ where, among 145 asymptomatic patients with very severe AS, the incidence of the composite of operative mortality or cardiovascular death was significantly lower among patients who underwent early AVR than patients who received conservative treatment. The ongoing EARLY TAVR (Evaluation of Transcatheter Aortic Valve Replacement Compared to Surveillance for Patients with Asymptomatic Severe Aortic Stenosis; NCT03042104) trial is enrolling 900 asymptomatic severe AS patients and randomizing them to early transcatheter AVR vs delayed valve replacement at symptoms onset. In moderate AS, the TAVR UNLOAD trial (Transcatheter Aortic Valve Replacement to Unload the Left Ventricle in Patients With Advanced Heart Failure; NCT02661451) is enrolling patients with moderate AS and reduced ejection fraction to investigate whether or not transcatheter AVR on top of optimal heart failure therapy improves clinical outcomes. Similarly, PROGRESS (Prospective, Randomized, Controlled Trial to Assess the Management of Moderate Aortic Stenosis by Clinical Surveillance or Transcatheter Aortic Valve Replacement; NCT04889872) and the EvolutTM EXPAND TAVR II Pivotal Trial (NCT05149755) are currently enrolling patients to demonstrate that transcatheter AVR may improve outcomes in patients with moderate AS who have symptoms or left ventricular dysfunction. The results of these trials will shed light on the great dilemma of early interventions in AS, probably further highlighting the existence of multiple nuances in the AS population.

In this rapidly evolving landscape, the use of ML algorithms, as proposed by Shimoni et al,^5^ enhances the ability to identify risk predictors to phenotype patients across the spectrum of AS, thus advancing the field in the direction of a holistic and patient-centered approach to AS.^5^

## Funding support and author disclosures

Dr Sannino has received grant support from Cardiovalve and Edwards Lifesciences. Dr Manzi has reported that she has no relationships relevant to the contents of this paper to disclose.
